# Oncoplastic breast conserving surgery versus standard breast conserving surgery for early and locally advanced breast cancer: a retrospective analysis from Sri Lanka

**DOI:** 10.1186/s12893-023-02182-5

**Published:** 2023-09-11

**Authors:** Kanchana Wijesinghe, Thilanka Abeywickrama, Yohan Chamara, Sumali De Silva, Sebastianpillai Tharshan, Umesh Jayarajah, Ajith De Silva

**Affiliations:** 1 Department of Surgery, Faculty of Medical Sciences, University of Sri Jayawardenapura, Nugegoda, Sri Lanka; 2 Department of Surgery, National Hospital of Sri Lanka, Colombo, Sri Lanka; 3 University Surgical Unit, Colombo South Teaching Hospital, Kalubowila, Sri Lanka

**Keywords:** Oncoplasty, Breast conserving surgery, Breast cancer, Oncosurgical outcomes, Aesthetic outcomes

## Abstract

**Background:**

Breast aesthetics is becoming increasingly important in breast cancer surgery due to changes in patient expectations and greater emphasis been placed on the psychosocial outcomes. Studies have shown no difference in local recurrence risk between mastectomy and breast conserving surgery (BCS) and also a higher overall survival rate after BCS. Breast preservation improves the quality of life substantially compared to mastectomy. Oncoplastic breast-conserving surgery (O-BCS) involves tumour excision whilst overcoming the limitations of standard breast conserving surgery (S-BCS) by allowing larger resection volumes, avoiding deformities with better aesthetic results. Our study aims to compare the oncosurgical and aesthetic outcomes of O-BCS versus S-BCS among women in Sri Lanka.

**Methods:**

We conducted a retrospective study over a 4-year period including patients who underwent breast conservation surgery for primary non-metastatic breast cancer in two tertiary care units. We assessed outcomes in terms of re-excision rates, resection margin, complications and aesthetic outcomes using a Likert scale questionnaire to grade specific outcomes such as symmetry, volume, nipple position, scar visibility. Non-parametric tests were used for statistical analyses.

**Results:**

Fifty-four and seventy-three patients underwent S-BCS and O-BCS respectively. The median specimen volume and the maximum tumour diameter were significantly higher in O-BCS [160(range:65–220); 4.2(range: 1.2–5.2)] compared to S-BCS [65(range:45–86); 2.4(range: 1.0-2.6)]. The median closest tumour margin was 16 mm (range:4-25 mm) in O-BCS while 6 mm (range:<1 – 12 mm) in S-BCS (p = 0.01). Close (< 1 mm) and positive margins needing re-excision were seen mostly in S-BCS. Superior aesthetic outcomes with statistical significant difference were reported in the O-BCS compared to S-BCS group with better symmetry, volume, nipple position and scar visibility. The re-excision rates were significantly lower in O-BCS group. There was no significant difference in the operative time and complications while the aesthetic outcomes were significantly superior in OBCS.

**Conclusions:**

Overall, Level 2 perforator flap based reconstruction had superior aesthetic outcomes. O-BCS is safe and more aesthetically acceptable with no difference in oncological outcome and operative time. More consideration should be given to aesthetic parameters such as scar visibility, nipple position, breast volume and shape when considering the best surgical option for the patients.

## Background

Breast cancer is the most common cancer among females in the world, affecting 12.5% of women [1]. Breast aesthetics is becoming increasingly important in breast cancer surgery due to changes in patient expectations and greater emphasis has been placed on the psychosocial and quality of life outcomes [2]. Studies have shown no difference in local recurrence risk between mastectomy and breast conserving surgery (BCS) and also a higher overall survival rate after BCS [3].

**Fig. 1:**
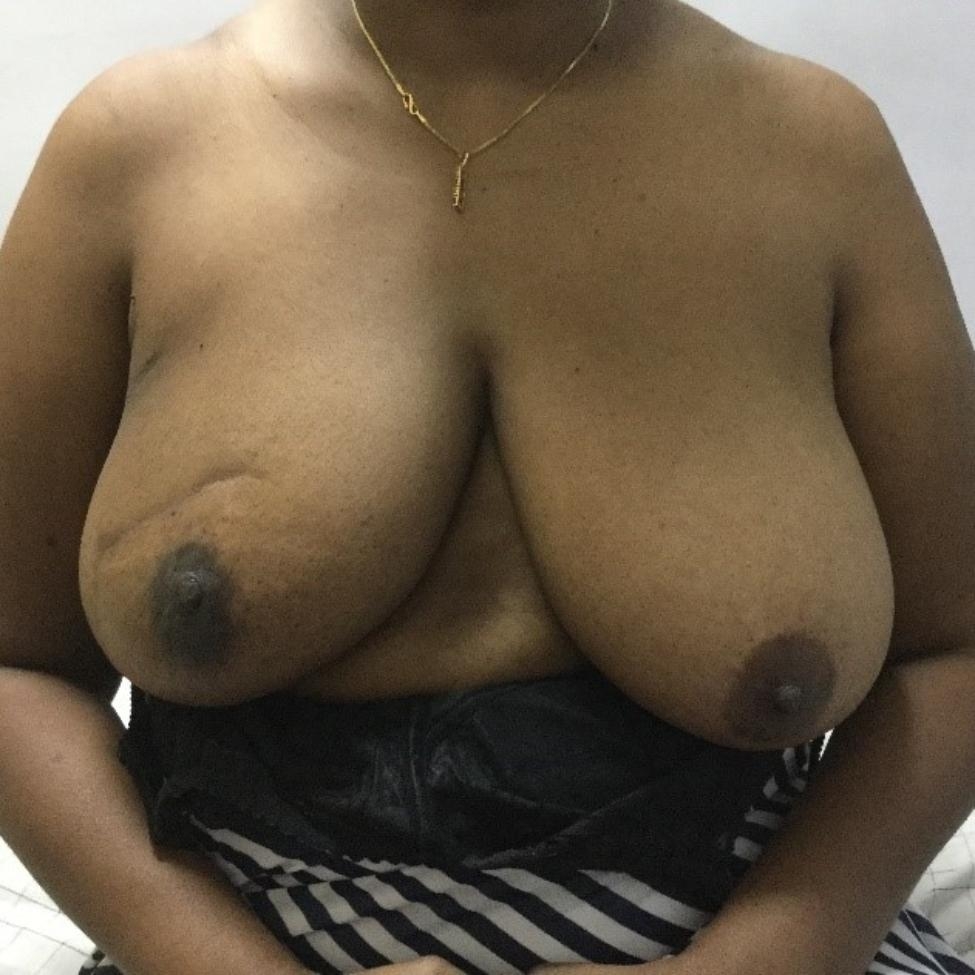
Patient who has undergone S-BCS with visible scar and difference in breast volume and nipple position

Literature shows that breast preservation improves the quality of life substantially compared to mastectomy, as it helps to maintain quality of life, preserves self-image, and positively impact on sexuality [4]. There are two main arms in BCS; the standard BCS and oncoplastic BCS. Standard breast-conserving surgery (S-BCS) is referred to performing a wide local excision applying the oncological principles and aims at removing enough breast tissue to ensure that the margins of the resected surgical specimen are free of tumor. Although S-BCS is safe and less mutilating than mastectomy, approximately 30% of the patients who undergo S-BCS are not satisfied with the aesthetic outcome [5, 6]. Therefore, balancing the oncologic need for wide local excision with the desire for an aesthetic result can be challenging in patients undergoing S-BCS (Fig. 1). These deformities and asymmetries have been described to contribute to negative body image and poor quality of life [7].

As a new paradigm in BCS, oncoplastic breast surgery (O-BCS) combines principles of oncology and reconstructive surgery towards achieving sound oncological and aesthetically pleasant results. Furthermore, O-BCS expands the indications for breast conservation allowing the resection of much larger tumours in relation to breast size. This approach reduces the incidence of positive margins and need for post-operative re-excision while preserving, or even enhancing, the natural shape, symmetry, and cosmetic appearance of the breast [8].

Breast cancer is the commonest cancer among females in Sri Lanka with a rising incidence [9]. However, there are very few studies regarding the aesthetic outcomes following breast cancer surgery in Sri Lanka and hardly any studies on the patient reported outcome measures (PROM) of the breast aesthetics [2]. There are many studies done in Western countries to describe the PROM. It would be educational to analyze the perception and attitudes of patients from a Sri Lankan socio cultural background and to improve on the delivery of breast cancer surgical care for the patients. It is in this background that this paper is aimed to describe the onco-surgical and aesthetic outcomes of BCS among women in Sri Lanka.

## Methods

A retrospective cohort study was conducted at the breast clinics of National Hospital of Sri Lanka and Colombo South Teaching Hospital in Sri Lanka including women with primary non metastatic breast cancer. All patients were discussed at the multi-disciplinary team (MDT) meeting and patients who were assigned to undergo breast conserving surgery during 2016 January to 2022 January were recruited.

All patients who were diagnosed with primary non metastatic breast cancer were initially assessed for suitability of breast conserving surgery at the MDT meeting. The stage of the breast cancer was decided on the clinical stage according to American Joint Committee on Cancer (AJCC) 7th edition traditional stage classification.

Several definitions of oncoplastic surgery have been reported in the literature. For our study purpose, we have utilized the classification system developed by the American Society of Breast Surgeons. It is divided into two levels: 20% volume loss as level 1 (which includes local tissue rearrangement) and 20–50% of breast tissue loss as level 2. The cut-off point of 20% separating levels 1 and 2 volume displacement oncoplastic surgery, and 50% separating volume displacement and volume replacement oncoplastic surgery, are fluid and should only serve as a planning guide. The choice of surgery should always be individualized to a patient’s cancer, breast, and personal priorities [10].

Aesthetic outcome is a primary outcome measure of breast conserving surgery. And research provides measurement tools to assess the aesthetic outcomes following breast conserving surgery such as the BREAST-Q or the Likert scale evaluation. Both these are validated scoring systems that measure how patients perceive their aesthetic and/or functional outcomes [11].

In our study, we used the aesthetic items scale assessment tool as the method for scoring aesthetic outcome after BCS. This tool has a 5-point Likert scale with respect to breast volume, shape, symmetry, scars, and nipple areola complex. For each of these items, a 5-point Likert scale is used for scoring. This scale ranges from “very dissatisfied,” “dissatisfied,” “neutral,” “satisfied,” to “very satisfied.” This scoring system is a validated and reliable method for evaluating the breast aesthetic outcomes [12, 13].

The data collection tool contained three arms. An interviewer administered questionnaire was used to collect socio-demographic and clinical data. This was a pre tested and modified version of a questionnaire that was previously used in a similar study [14].

Second component consisted of the onco-surgical outcomes that were by re-excision rates, resection margin and complications according to the Clavien-Dindo classification [14]. The third part of the questionnaire included the aesthetic assessment using a Likert scale questionnaire to grade specific outcomes such as symmetry, volume, nipple position, scar visibility [12, 13]. Two independent assessors (senior surgical medical officers) were used for aesthetic outcome assessment at six months and one year following surgery. At the same visit, the patients were also invited to assess the aesthetic outcome using the questionnaire independently.

A comparative analysis was performed on the aesthetic outcomes between the two main arms, the type of oncoplastic surgery and age. Comparison of these categorical variables was performed using the Pearson Chi square test. A p value of < 0.05 was be considered as statistically significant.

Data processing and analysis was done using SPSS statistical software ver.19. Categorical variables were presented using descriptive statistics like frequency and percentages. Continuous variables were expressed in terms of mean and standard deviation. Associations between continuous variables were explored using Pearson’s correlation.

## Results

### Patient and tumour characteristics

A total of 127 patients had undergone breast conserving surgery during the study period, of which 47.5% have been offered oncoplastic reconstruction. The median age and the range was lower in the O-BCS arm although this was not statistically significant. In both arms, the majority of the tumors were located in the upper outer quadrant (UOQ).

In terms of TNM staging, both S-BCS and O-BCS had T1 and T2 stage tumors, with a majority T1 and in situ stage tumors being offered S-BCS. Around 59.7% (46/77) of T2 tumours were in O-BCS arm. All T3 stage tumors were in O-BCS arm. None of the tumours were of stage T4. There were 7 patients with T1 lesions in the O-BCS arm of which 5/7 were in the upper inner quadrant (UIQ) and 2/7 in the lower inner quadrant (LIQ). This could be attributed to the small volume in the inner quadrants requiring an oncoplastic surgery. Twenty-one patients (16.5%) received neo-adjuvant chemotherapy and the post chemotherapy tumour size was taken as the tumour stage at surgery. There was a statistical difference in the median tumour size in both arms with much larger tumor size being offered O-BCS. (Table 1)

**Table 1:** Patient and tumor characteristics of the study group

Variable	S-BCS n (%) median and range	O-BCS n (%) median and range	p value
Patients	54(42.5%)	73 (47.5%)	
Age (years)	56 (42–74)	51 (39–68)	0.055
Histological Tumour size	2.4 (1–2.6)	4.2 (1.2–5.2)	0.041
Multifocality	00	05	-
Tumour localization – central	09	14	0.716
- upper inner quadrant	04	09	0.365
- upper outer quadrant	22	30	0.835
- lower inner quadrant	07	12	0.587
- lower outer quadrant	12	08	0.084

### Surgical treatment

The median specimen volume was significantly higher in OBCS as well as the closest margin. The operative time was prolonged in the O-BCS arm however, this was not statistically significant. Re excisions were mostly seen in S-BCS. There was one patient in the O-BCS arm that required re-excision due to presence of ductal carcinoma in-situ (DCIS) in the margin. A variety of O-BCS techniques were offered to patients as illustrated in Table 2. The only complication noted was wound dehiscence which was Clavien Dindo Grade 1. (Table 3)

**Table 2:** Types of Oncoplastic breast surgery performed in the study (intercostal artery perforator flap-ICAP; Lateral thoracic artery perforator flap- LTAP; Thoracodorsal artery perforator flap- TDAP)

Type of surgery	Number
Level 1(Volume displacement)	
Crescent mastopexy	05
Batwing resection	09
Donut mastopexy/round block technique	04
Central quadrantectomy	05
Level 11 (Volume displacement)	
Burow’s triangle advancement flap (Fig. 2)	10
J-mammoplasty	03
Thoraco-lateral advancement flap	08
Bilobed flap (Fig. 3)	03
Reduction mammoplasty	04
Level 11 (Volume replacement)	
Chest wall artery based perforator flaps (ICAPs/LTAP) (Figs. 4 and 5)	22

**Table 3:** Surgical outcomes following BCS

Variable	S-BCS n (%) or median/range	O-BCS n (%) or median/range	p value
Median specimen volume	65 (45–86) ml	160 (65–220) ml	< 0.05
Closest tumour margin	6 mm (< 1–12)	16 mm (4–25)	< 0.05
Re-excision for affected surgical margins	09 (16.7%)	01 (1.4%)	< 0.01
Operative time (minutes)	58 (47–90)	67 (55–96)	0.794
Complications -			
• Wound dehiscence	01	03	-
• Infection	00	00	-

**Fig. 2:**
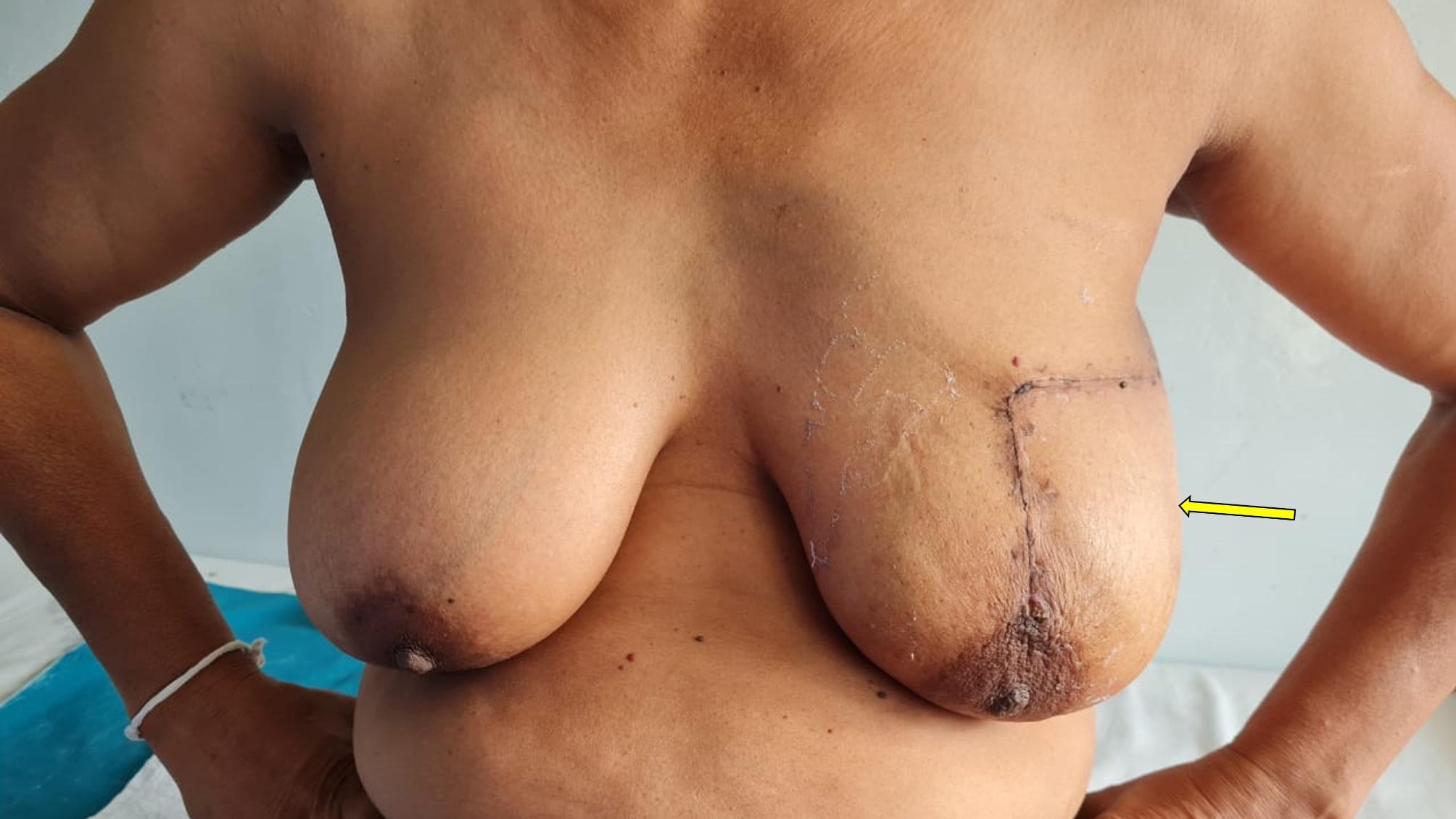
A patient who has undergone O-BCS with matrix rotation technique. Although the scar is visible, this technique helps to maintain breast shape, symmetry and nipple position

**Fig. 3:**
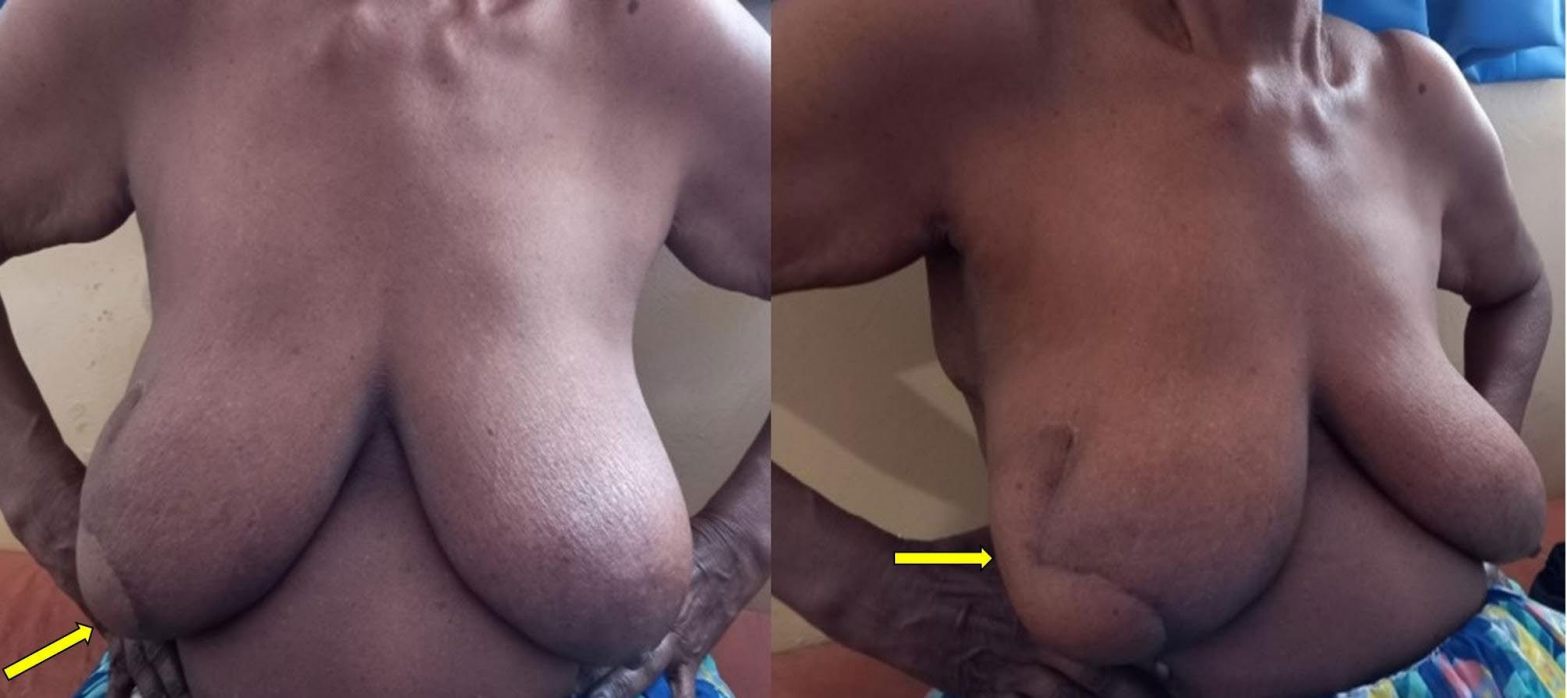
Bi-lobed flap based O-BCS for a patient who declined reduction mammoplasty and wished to retain the breast shape and size

**Fig. 4:**
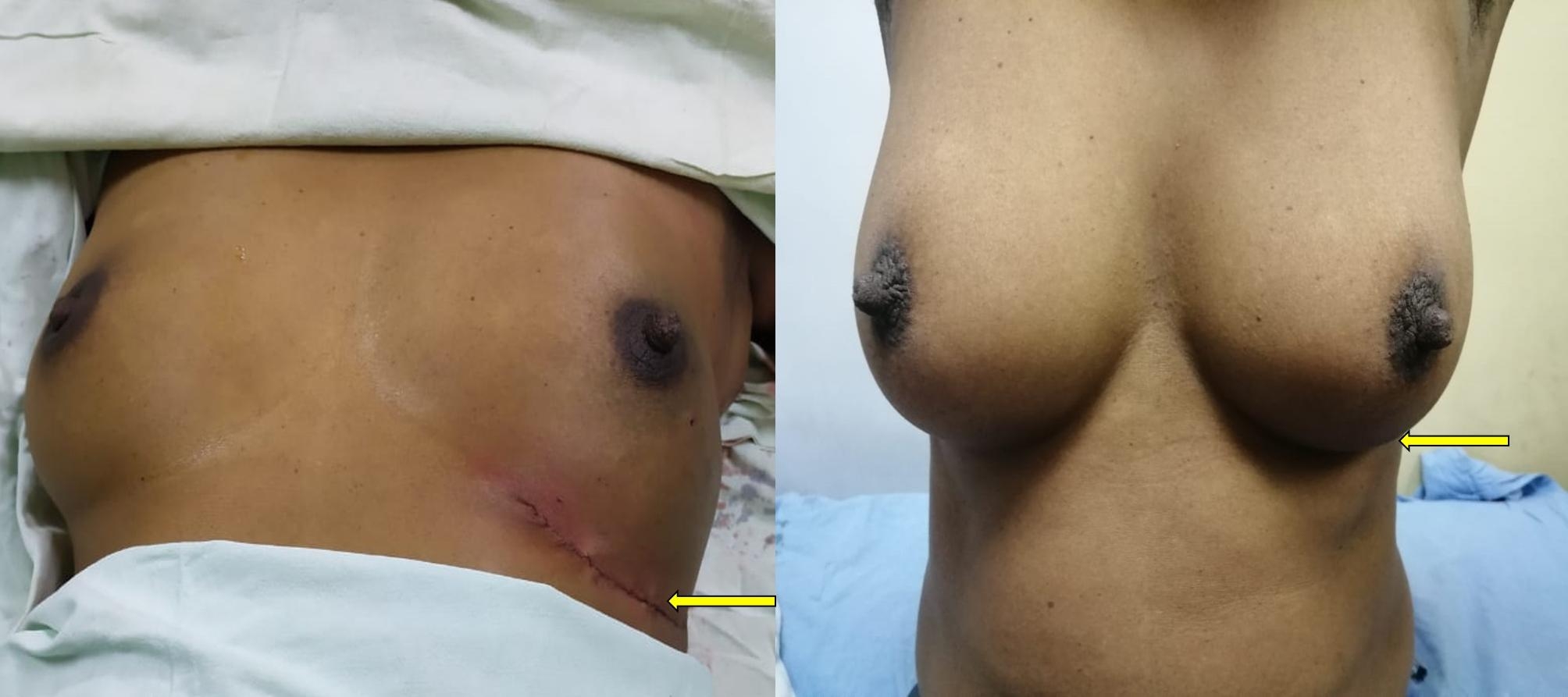
Intraoperative and post-operative (6 months) images of a patient who underwent LICAP flap based O-BCS. There is minimal scar visibility with symmetrical breast volume and shape

**Fig. 5:**
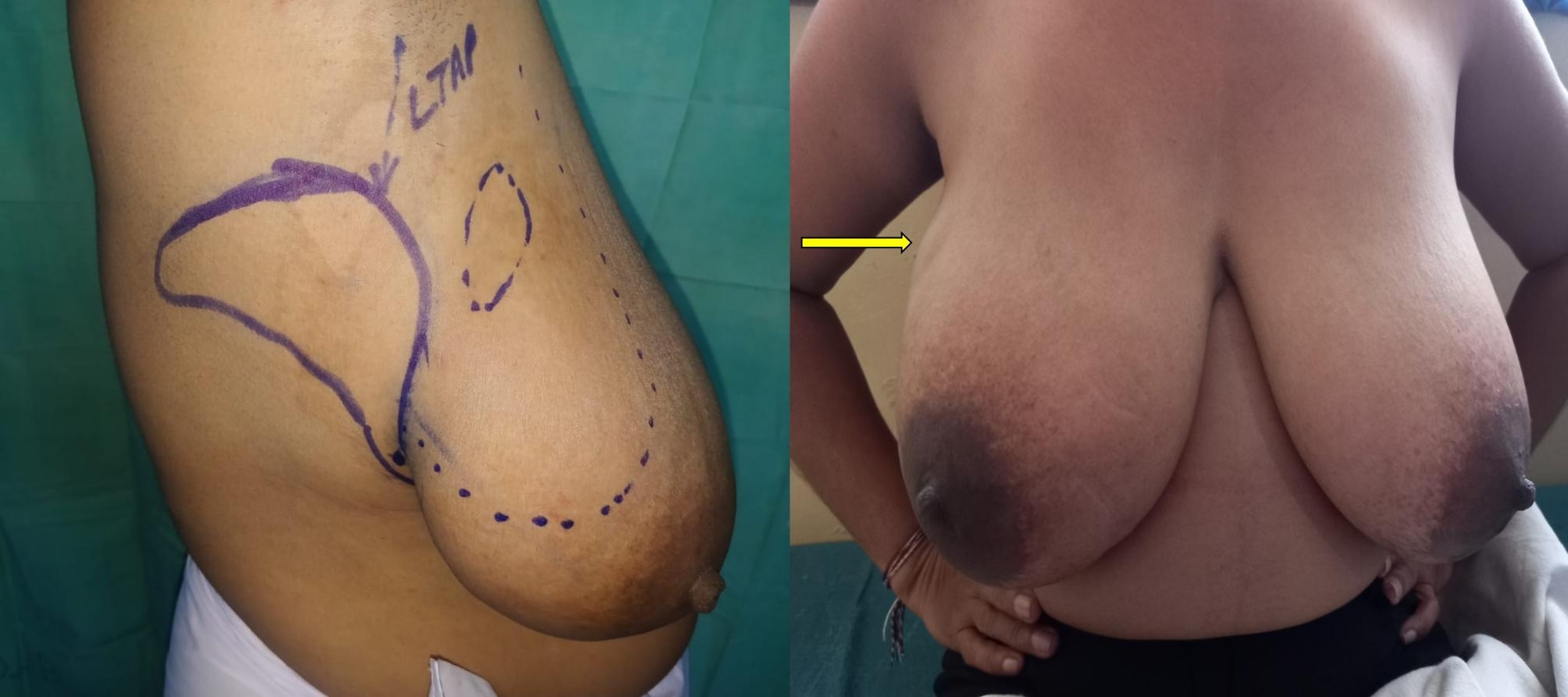
Pre-operative and post-operative (6 months) images of a patient who underwent LTAP flap based O-BCS. There is minimal scar visibility with symmetrical breast volume and shape

### Types of O-BCS performed in our study

The O-BCS study arm consisted of 73 patients, of which 31.5% underwent Level 1 oncoplastic surgery. Table 2 details the various types of surgical techniques performed under the umbrella of O-BCS.

Chest wall perforator flaps were performed in 22 patients in the O-BCS group. Lateral intercostal artery perforator (LICAP), anterior intercostal artery perforator (AICAP), medial intercostal artery perforator (MICAP) and Lateral thoracic artery perforator (LTAP) flap were performed in 12 (54.5%), 3 (13.6%), 2 (9.1%) and 2 (9.1%) patients, respectively. 3 (13.6%) patients had combined LTAP and LICAP. Lateral quadrant defects were reconstructed by LICAP and LTAP flap or a combination the of two, and medial and 6 0 clock position defects were reconstructed by the AICAP or MICAP flap. (Figures 4 and 5)

### Comparison of aesthetic assessment of S-BCS versus O-BCS

**Fig. 6:**
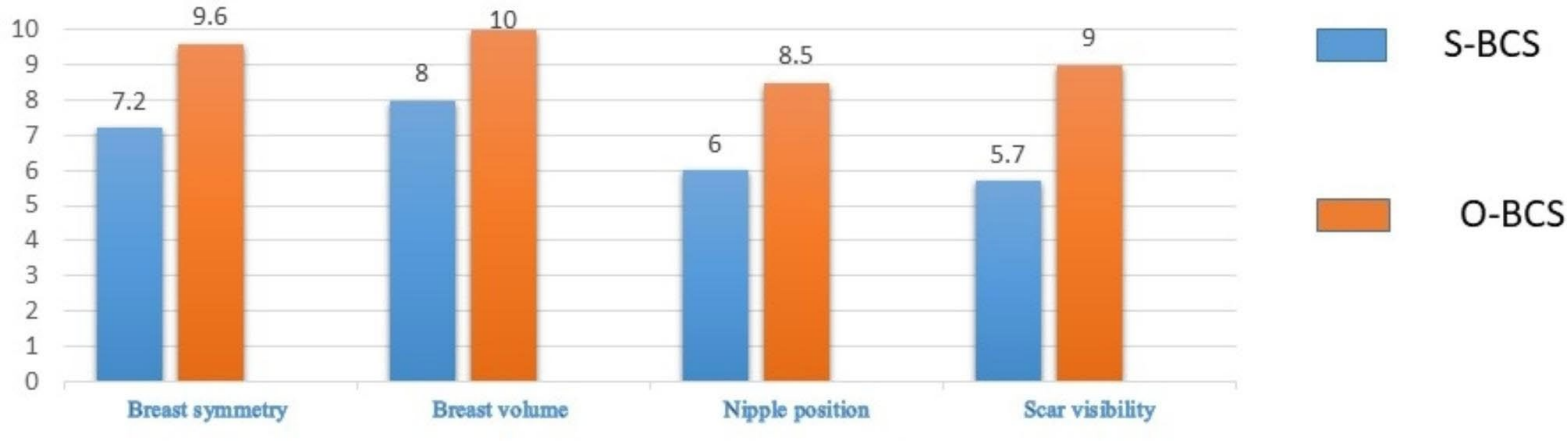
Comparison of the median values of the aesthetic outcome – Independent specialist assessment at one year

**Fig. 7:**
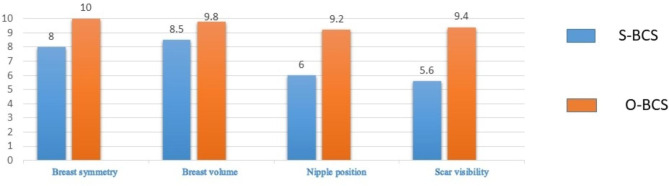
Comparison of the median values of the aesthetic outcome – Patient reported outcome measurement at one year

Aesthetic items scale assessment tool was used to score the aesthetic outcome after BCS. The assessment was obtained by an independent medical practitioner as well as the patients. A statistically significant difference was seen in the aesthetic outcome on nipple position and scar visibility in the two arms, with O-BCS showing overall better aesthetic outcome. This observation is noted in both the independent specialist assessment as well as in the patient reported outcomes. (Figures 6 and 7)

### Comparison of the PROM aesthetic outcome based on the type of oncoplastic surgery

We analyzed if there was a difference in the patient reported aesthetic outcome depending on the level of oncoplastic surgery performed (Figs. 4, 5, 2 and 3). We observed superior aesthetic outcome was with level 2 volume displacement perforator flaps techniques. Patients reported a 10/10 for scar visibility and nipple position for perforator flaps surgeries. (Table 4)

**Table 4:** Median value of the PROM aesthetic outcome based on the type of oncoplastic surgery

Type of oncoplastic surgery	Breast volume	Breast symmetry	Nipple position	Scar visibility
**Level 1**	7.6	6.8	7.4	6.6
**Level 2**				
Volume displacement flaps	8.6	8.4	8	8.8
Volume replacement flaps ICAP/LTAP flaps	9.6	9.8	10	10

### Comparison of aesthetic outcome in relation to age and type of surgery

**Fig. 8:**
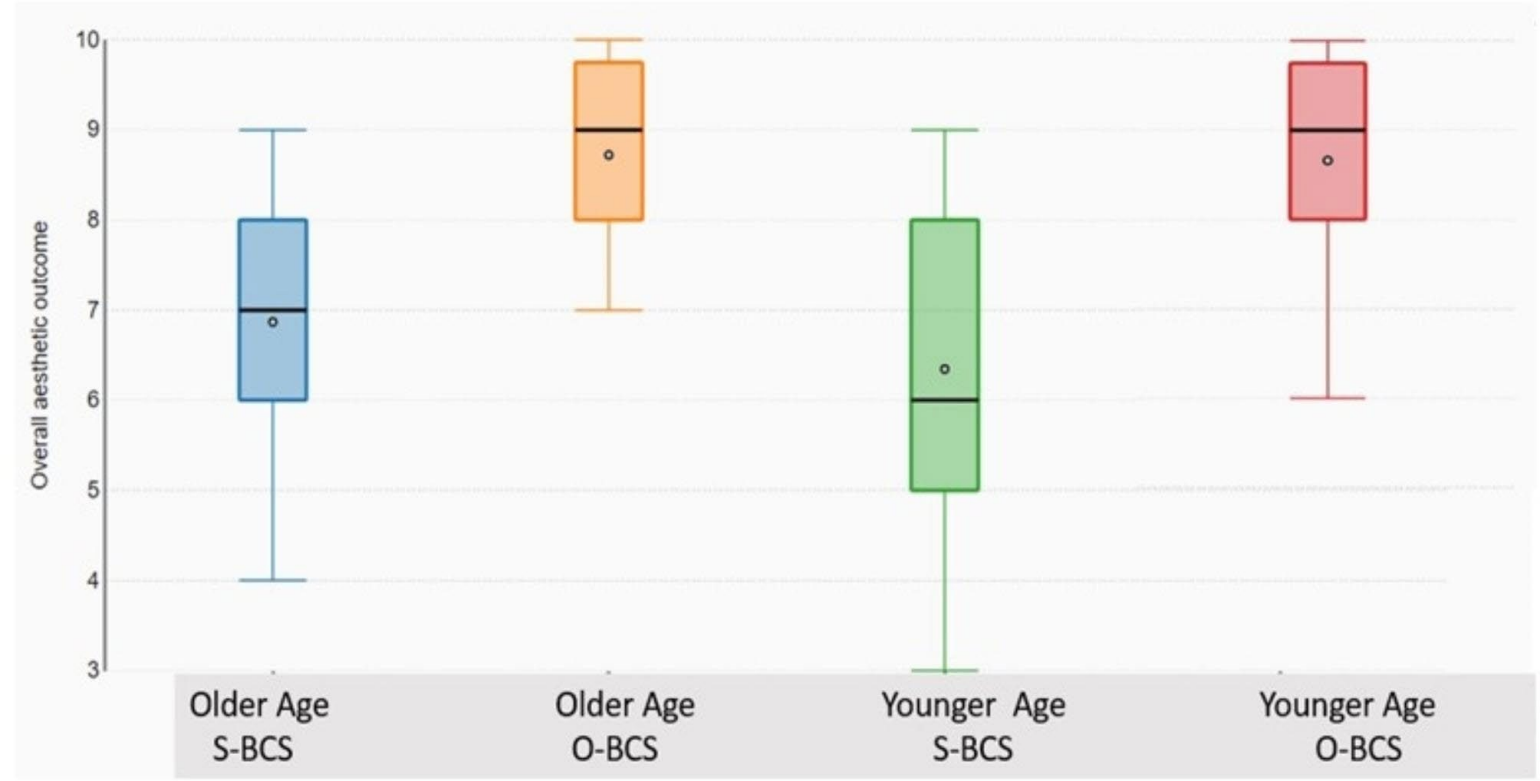
Box plot comparing the median values of the aesthetic outcomes in relation to age and type of surgery

Patients were categorized as less than or equal to 60 years (younger) and more than 60 years (older) according to the age threshold defined by the United Nations. In our study, 62% (n = 79) belonged to the younger age category. O-BCS was offered to 63.3% in the younger age group and 37.5% in the older age group respectively. In both age groups, O-BCS showed better aesthetic outcome compared to the standard arm. (Fig. 8)

## Discussion

In this study, we report the onco-surgical and aesthetic outcomes of a cohort of 127 patients who underwent BCS for breast cancer in Sri Lanka.

The patients were categorized according to the type of BCS performed and 47.5% of patients had been offered O-BCS. Both groups were homogenous in terms of numbers and the age range. In comparison to similar studies, we have a higher recruitment to O-BCS group compared to S-BCS group [15]. One plausible reason is that many studies were done in the Western population where breast cancer is commonly diagnosed at T1 stage which is amenable for S-BCS. In Sri Lanka, most of the patients belong to the T2 or T3 stage requiring the need for more O-BCS [16]. In our study, 76.3% (n = 97) belonged to T2 and T3 staged tumors. This also highlights the fact for the need of more robust breast cancer detection programmes in Sri Lanka [17, 18].

We also noted that in both arms, the majority of the tumors were located in the UOQ, which is a well-established observation in literature. The commonly accepted explanation for this observation is that greater amount of breast tissue is situated in the UOQ [19].

Of the seven patients with T1 lesions in the O-BCS group, five of them had the tumor located in the UIQ. Wide excision in the UIQ leads to significant deformities and even visible scars. Thus, careful attention has to be given when planning BCS in the UIQ. Grisotti et al. defined the upper inner quadrant as the “no man’s land” due to these challenges faced in performing BCS [20]. In our study, we too were cautious in the surgical decision making for UIQ tumors. All these patients underwent O-BCS either with Batwing incision or Burow’s triangle (Matrix rotation) techniques (Fig. 2) to obtain better aesthetic outcomes. These techniques are widely used in similar studies and have shown to produce good surgical outcomes [21].

A striking feature in our study is that the operative time between both groups did not show a statistically significant difference although in O-BCS group the operative time was comparatively higher. To our knowledge, this is one of the first studies that has compared the operative time in the two arms. This data suggests the O-BCS although technically advanced than S-BCS, does not take significant additional time to perform and does not have significantly higher complication rates. In the Sri Lankan setting with limited operating theatre time and with no dedicated theatre for breast surgery, performing O-BCS will not have a major impact on the theatre list. Re-excisions were mostly seen in S-BCS as seen in literature. There was one patient in the O-BCS arm that required re-excision due to presence of DCIS in the margin. Increased resection margins have not been shown to improve the oncological safety and the primary purpose of O-BCS is not to obtain better margins [22, 23]. However, this has the added benefit of significantly lowering the re-excision rates, which is clearly observed in our study and similar studies as well.

In this study, a variety of oncoplastic surgical techniques have been used to treat patients. It is interesting to note that the aesthetic outcome assessment at one year is significantly superior in the Level 2 O-BCS in comparison to Level 1 O-BCS, in terms of scar visibility and nipple position. This observation is noted in both the independent specialist assessment as well as in the patient reported outcomes. The aesthetic outcome assessment of breast symmetry and volume also have better results with Level 2 O-BCS. We assessed whether age could affect the patients’ aesthetic appreciation of the breast but both age groups showed superior outcomes in the O-BCS arm. These observations led us to sub analyze the aesthetic outcomes of Level 2 O-BCS arm. Superior outcomes were observed mainly in the perforator flap based reconstructions (Table 4).

Chest wall perforator flaps were initially described by Hamdi et al. as perforators arising from the deep vascular system through the underlying muscles or intermuscular septum [24]. Since then many studies have been done in the last two decades describing the versatility of chest wall perforator flaps for partial breast reconstruction following wide local excision [25].

In chest wall perforator flap based reconstructions, the surgical incision is hardly visible as it is placed in the infra mammary or lateral mammary fold (Figs. 4 and 5). This also in turn could minimize nipple deviation as a result of surgical incision. These perforators enable harvesting of large flaps that are equal or more than the volume resected. This helps to retain the shape and volume of the breast. One main concern is flap shrinkage and reduction in breast volume in the future. On one-year assessment in our study, a notable volume shrinkage was observed only in three patients involving the anterior intercostal artery perforator (AICAP) (n = 2) and medial intercostal artery perforator (MICAP) (n = 1). The results of the perforator flap reconstruction are comparable to the existing data.

This study is among the few studies that have compared the aesthetic outcome of different oncoplastic techniques. Although a deeper analysis of the perforator flap based reconstruction is beyond this article, it is worth noting the superior outcomes of perforator flap based reconstruction and future comparative studies are recommended.

### Study limitations

We did not analyze the breast to tumor volume ratio as this data was not available in the retrospective analysis. The effect of adjuvant therapy on breast conserving surgery was not considered. Usage of a more robust method for aesthetic assessment like Breast Q would have given a more details regarding the aesthetic outcome [26]. However, we chose a simpler method as it was more feasible in our population.

## Conclusion

Our study group consisted of 127 patients diagnosed with breast cancer of varying tumour stages who underwent BCS. The re-excision rates were significantly lower in O-BCS group. There was no significant difference in the operative time and complications while the aesthetic outcomes were significantly superior in OBCS. Overall, Level 2 perforator flap based reconstruction had superior aesthetic outcomes. O-BCS is safe and more aesthetically acceptable with no difference in oncological outcome and operative time. More consideration should be given to aesthetic parameters such as scar visibility, nipple position, breast volume and shape when considering the best surgical option for the patients.

## Data Availability

The data used in the above analysis will be available on reasonable request from the corresponding author.

## List of abbreviations

AICAP: Anterior intercostal artery perforator
BCS: Breast conserving surgery
DCIS: Ductal carcinoma in situ
LIQ: Lower inner quadrant
MDT: Multi-disciplinary team
MICAP: Medial intercostal artery perforator
O-BCS: Oncoplastic breast-conserving surgery
PROM: Patient reported outcome measures
S-BCS: Standard breast conserving surgery
SPSS: Statistical package for social sciences
UIQ: Upper inner quadrant
UOQ: Upper outer quadrant
